# Sustaining Muscle, Cardiovascular Health, and the Environment: Is Plant-Based Protein the Key?

**DOI:** 10.3390/nu18091446

**Published:** 2026-04-30

**Authors:** Teresa Cannito, Alja Ivetac, Nicola Fiotti, Filippo Mearelli, Stefano Di Bella, Filippo Giorgio Di Girolamo, Gianni Biolo

**Affiliations:** 1 Clinica Medica Unit, University of Trieste, 34128 Trieste, Italy; t.cannito95@gmail.com (T.C.); alja.ivetac@gmail.com (A.I.); fiotti@units.it (N.F.); filippo.mearelli@asugi.sanita.fvg.it (F.M.); 2 Clinical Department of Medical, Surgical and Health Sciences, Ospedale di Cattinara, 34149 Trieste, Italy; stefano.dibella@asugi.sanita.fvg.it; 3 Infectious Diseases Unit, University of Trieste, 34128 Trieste, Italy; 4 Pharmacy Unit, University of Trieste, 34128 Trieste, Italy; fgdigirolamo@units.it

**Keywords:** muscle, aging, environment, plant-based proteins, animal-based proteins

## Abstract

**Background/Objectives**: Protein intake is a key determinant of skeletal muscle health across the lifespan, yet optimal strategies must also account for cardiometabolic health and environmental sustainability. Differences in digestibility and amino acid composition between plant and animal-based proteins may influence their capacity to stimulate muscle protein synthesis (MPS), particularly in aging. **Methods**: This narrative review integrates evidence from acute tracer studies, randomized controlled trials, and long-term observational research comparing plant versus animal-based proteins for preserving muscle while supporting environmental goals and cardiometabolic health across populations. PubMed and Google Scholar were searched from inception to 11 December 2025 (plant-based protein OR animal-based protein AND sarcopenia OR muscle protein synthesis), with citation tracking. In total, 80 relevant findings were identified. **Results**: Acute tracer studies show that, gram-for-gram, animal-based proteins (particularly whey/dairy) stimulate greater myofibrillar protein synthesis due to higher leucine density, digestibility, and more rapid aminoacidemia—an effect that is more pronounced in older adults with anabolic resistance. In younger individuals, these differences are largely attenuated when total protein intake is sufficient. Importantly, the anabolic potential of plant-based proteins can be enhanced through higher dosing, amino acid or leucine fortification, and complementary protein blending (e.g., cereals with legumes or use of high-DIAAS isolates). Consistent with this, longer-term resistance training studies demonstrate comparable gains in muscle mass and strength between plant- and animal-based diets when protein intake (≥1.0–1.2 g/kg/day; ≥1.2–1.5 g/kg/day in illness), per-meal distribution (~0.4 g/kg with ~3–4 g leucine in older adults), and energy intake are optimized. Beyond muscle outcomes, higher plant-based protein intake is associated with favorable cardiometabolic profiles and lower environmental impact. **Conclusions:** An age-specific, mixed protein approach is recommended, emphasizing plant-based proteins in younger adults and higher-quality, leucine-rich proteins in older individuals. Defining optimal plant-to-animal-based protein ratios remains a key research priority.

## 1. Introduction

Preservation of skeletal muscle mass and function across the lifespan is a central determinant of metabolic health, physical independence, and overall quality of life. However, aging is accompanied by a progressive decline in muscle mass and anabolic sensitivity, a phenomenon often referred to as anabolic resistance, which is further exacerbated by physical inactivity and disuse [1,2,3]. This condition leads to an impaired muscle protein synthetic (MPS) response to dietary protein and contributes to the development of sarcopenia [1,4,5,6,7]. Accordingly, optimizing protein nutrition has emerged as a key strategy to maintain muscle health, particularly in older adults [2,8,9].

At the same time, dietary recommendations are increasingly shaped not only by physiological requirements but also by cardiometabolic health and environmental sustainability. Large prospective cohort studies consistently report that higher intakes of plant-based proteins are associated with lower cardiovascular and all-cause mortality, whereas higher consumption of certain animal-based protein sources—particularly processed red meat—is linked to adverse health outcomes [10,11,12,13,14,15]. In parallel, the environmental impact of animal agriculture, especially ruminant meat production, has prompted a shift toward more plant-forward dietary patterns due to their substantially lower greenhouse gas emissions and resource use [10,11,16].

This dual challenge—preserving muscle mass in an aging population while reducing the negative health and environmental impacts of high animal-protein diet raises a central question: can plant-based protein be the key to both sustaining muscle and sustaining the environment? However, important physiological differences between protein sources must be considered. Animal-based proteins are generally characterized by higher digestibility (≈85–95%), greater essential amino acid (EAA) density, and higher leucine content, all of which are key determinants of postprandial MPS [17,18,19]. In contrast, many plant-based proteins exhibit lower digestibility (~50–75%) and less optimal amino acid profiles, although these limitations can be mitigated through strategies such as protein blending, fortification, and increased intake [17,18,19].

Within this context, a key unresolved question is how to appropriately balance plant and animal-based protein intake across the lifespan to support muscle health while also considering cardiometabolic outcomes and environmental sustainability.

The present narrative review addresses the following issues: age-related anabolic resistance and protein requirements; digestion, absorption and quality of animal- versus plant-based proteins; acute muscle protein synthesis (MPS) responses to plant and animal-based proteins; long-term effects of protein sources on muscle mass and function; cardiometabolic and environmental implications of shifting protein sources. In addition, practical strategies for designing plant-forward dietary patterns that preserve muscle in older adults are discussed.

Accordingly, the main objectives of this work are to (i) evaluate how the relative contribution of plant and animal-based proteins influences muscle and cardiometabolic health across different age groups, as well as environmental sustainability, and (ii) compare the protein requirements and anabolic responses between young and older adults in order to better inform age-specific nutritional strategies, rather than a one-size-fits-all approach.

## 2. Materials and Methods

This narrative review was conducted to synthesize the available evidence on the role of plant and animal-based proteins in the regulation of muscle protein synthesis, muscle mass, and function across the lifespan, with additional consideration of cardiometabolic and environmental implications. As a narrative review, it does not follow a formal systematic protocol; rather, it integrates evidence from multiple study designs, including randomized controlled trials, stable isotope tracer studies, meta-analyses, prospective cohort studies, and mechanistic investigations, with the aim of providing a comprehensive and critically appraised overview of the field. PubMed and Google Scholar were searched from inception up to 11 December 2025 using the following key search terms: (plant-based protein OR animal-based protein) AND (sarcopenia OR muscle protein synthesis). The search yielded a total of 129 records. After careful screening and evaluation of all retrieved articles, 80 studies were deemed relevant and included in this narrative review. The search was restricted to articles published in English and conducted in humans aged ≥ 18 years. Studies involving clinical populations, older adults, and healthy young adults were all considered relevant to the specific topic addressed. Priority was given to studies employing stable isotope tracer methodologies, the gold standard for the in vivo quantification of postprandial myofibrillar protein synthesis rates, as well as to randomized controlled trials and meta-analyses examining long-term effects of protein source on muscle mass and function. Observational and epidemiological studies were included, particularly for cardiometabolic and environmental outcomes. The review is organized into the following sections: mechanism of anabolic resistance, protein requirement and the impact of exercise; protein digestion, absorption, and quality across animal and plant sources; acute muscle protein synthetic responses to plant versus animal-based proteins; long-term effects of protein source on muscle mass and function; cardiometabolic and environmental implications of shifting protein sources and practical implications for designing plant-forward diets that preserve muscle health.

## 3. Regulation of Muscle Protein Synthesis at Rest and After Exercise in Young and Older Adults

Aging and physical inactivity are potent inducers of anabolic resistance. Protein ingestion to stimulate myofibrillar protein synthesis requires greater relative protein intakes in healthy older versus younger subjects [2]. In a two-week bed rest model, older men show a greater decline in post-prandial muscle protein anabolic response and larger losses of muscle mass compared with younger adults, despite identical nutritional support [3]. Experimental bed rest and disuse consistently reduce post-absorptive MPS and blunt the MPS response to protein ingestion or mixed meals, thereby accelerating muscle atrophy [1,3]. At the molecular level, anabolic resistance reflects impaired translation initiation (e.g., reduced mTORC1 signaling), diminished muscle perfusion and altered amino acid transport and intracellular sensing. Chronic low-grade inflammation, insulin resistance, ectopic fat accumulation and mitochondrial dysfunction further compromise anabolic signaling in older muscle [1,2]. In view of these changes, multiple expert groups have proposed higher protein intakes for older adults than those recommended for younger populations. The PROT-AGE Study Group suggests 1.0–1.2 g/kg/day for healthy older adults and ≥1.2–1.5 g/kg/day in those with acute or chronic illness, provided renal function is adequate [8]. ESPEN experts similarly recommend ≥1.0–1.2 g/kg/day, emphasizing higher intakes during illness, rehabilitation and sarcopenia [9]. Equally important is the distribution and quality of protein across meals. Experimental data indicate that MPS is maximally stimulated by ~0.25–0.3 g/kg of high-quality protein per meal in younger adults and ~0.4 g/kg/meal in older adults, corresponding to ~20–25 g and ~30–40 g of high-quality protein per meal, respectively [2,10]. The concept of a leucine “trigger” highlights that ~2–3 g leucine per meal in younger adults and ~3–4 g in older adults are required to robustly activate mTORC1 and MPS [20,21]. Leucine is a key regulator of translational initiation and acts as a nutrient signal for mTORC1. Studies in older adults show that increasing the proportion of leucine within an essential amino acid mixture enhances MPS, even when total amino acid dose is unchanged [20]. Supplementation of suboptimal protein doses with free leucine or essential amino acids can restore the anabolic response in younger adults, and chronic leucine or EAA supplementation may modestly improve lean body mass and function in older individuals with low baseline protein intake [21,22,23]. These values represent pragmatic heuristics derived from controlled acute studies, rather than universal cut-offs. In practice, the leucine dose required to activate MPS varies with age, protein intake, protein source, exercise status and mixed meal composition. Therefore, it should be applied as target within a dietary strategy rather than a precise threshold.

Resistance exercise increases skeletal muscle sensitivity to essential amino acids for several hours (and up to 24 h), so ingesting leucine-rich protein bolus (≈20–40 g, depending on body size/age) after training amplifies mTORC1-dependent translation initiation and supports myofibrillar remodeling [24,25,26,27]. Co-ingesting carbohydrate raises insulin, which improves amino-acid delivery and supports net anabolism [28]; however, insulin elevations add little to MPS if protein/leucine provision is already adequate [28,29,30]. In mixed meals, insulin also facilitates muscle perfusion and amino-acid transport, reinforcing the post-resistance “anabolic window” driven by amino acids.

**Figure 1:**
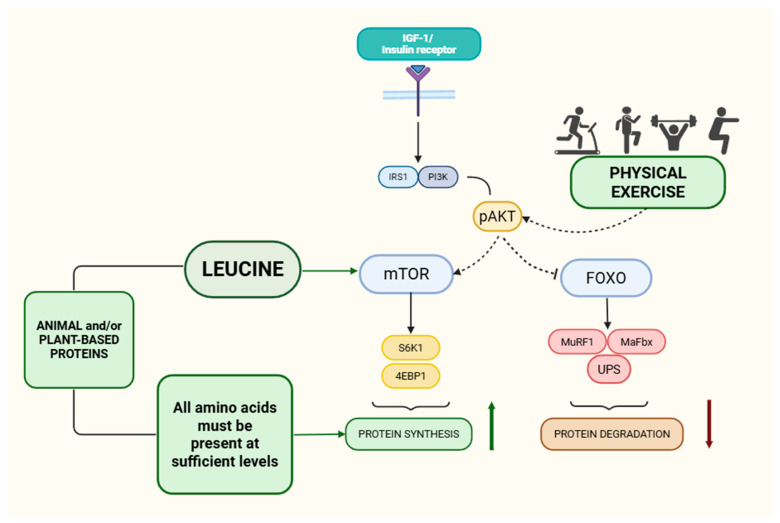
Integrated regulation of skeletal muscle protein turnover by resistance/aerobic exercise and nutrient–hormonal signals. The schematic depicts convergence of IGF-1/insulin signaling and exercise-derived cues on key anabolic and anti-catabolic nodes controlling muscle protein synthesis and degradation. Binding of insulin/IGF-1 to its receptor activates IRS1 and PI3K, leading to Akt phosphorylation (pAkt). Activated Akt promotes anabolism partly by facilitating mTORC1 signaling and by inhibiting FOXO transcription factors. In parallel, resistance and aerobic exercise provide contractile and metabolic stimuli that can enhance Akt signaling and, through mechanotransduction, directly support mTORC1 activation. Leucine acts as a nutrient signal that amplifies mTORC1 activity [36]; however, sustained protein synthesis requires adequate availability of essential amino acids, which provide the necessary substrate for translation. Downstream of mTORC1, phosphorylation of S6K1 and 4E-BP1 promotes translation initiation and increases protein synthesis. Conversely, FOXO activation induces “atrogenes” encoding E3 ubiquitin ligases (e.g., MuRF1 and MAFbx/Atrogin-1), stimulating the ubiquitin–proteasome system (UPS) and increasing proteolysis [37]. Thus, the net post-exercise anabolic response reflects coordinated upregulation of translational machinery via mTORC1 together with suppression of proteolytic gene programs via Akt-FOXO inhibition, contingent on sufficient essential amino acid supply [38]. Abbreviations: IGF-1, insulin like growth factor 1; IRS-1, insulin receptor substrate-1; PI3K, phosphoinositide 3-kinase; Akt, protein kinase B; mTORC1, mechanistic target of rapamycin complex 1; S6K1, ribosomal protein S6 kinase 1; 4EBP1, eukaryotic initiation factor 4E-binding protein 1; FOXO, forkhead box O; MuRF1, muscle RING-finger protein-1; MAFbx, muscle atrophy F-box; UPS, ubiquitin–proteasome system. Animal-based proteins are more easily digestible and characterized by a more rapid and efficient increase in circulating essential amino acids, reaching the leucine threshold more quickly. In contrast, plant-based proteins tend to have lower digestibility and a different amino acid profile, requiring higher intake or fortification or complementary sources to achieve a comparable leucine threshold. Nevertheless, when combined with exercise, both protein sources can effectively stimulate muscle protein synthesis and support anabolic response.

Aerobic exercise also stimulates MPS—particularly mitochondrial protein synthesis—and can augment insulin sensitivity and microvascular perfusion, facilitating amino-acid delivery and attenuating anabolic resistance when protein is provided post-exercise and/or distributed across meals [31,32,33]. Concurrent (resistance + aerobic) training generally supports both hypertrophy and oxidative adaptations when protein intake is sufficient, although very high endurance volumes may shift cellular priorities toward endurance-linked signaling (e.g., AMPK-associated pathways), making total daily protein and per-meal leucine content more important to preserve myofibrillar accretion [25,26,34,35]. In practise, timing protein ingestion near exercise sessions and ensuring adequate daily protein intake to match training load maximizes the integrated anabolic response and supports both contractile and mitochondrial remodeling [24,25,26,31,32,33,34,35] (Figure 1).

## 4. Protein Digestion, Absorption and Quality: Animal Versus Plant Sources

The muscle protein synthetic response to protein ingestion varies substantially between dietary protein sources and is largely determined by the postprandial rise in circulating essential amino acid (EAA) concentrations, particularly leucine. The magnitude and duration of aminoacidemia—and the subsequent stimulation of MPS—are influenced by multiple physiological processes, including protein digestion, amino acid absorption, splanchnic extraction, tissue perfusion, and skeletal muscle amino acid uptake, as well as activation of intracellular anabolic signaling pathways [25,39,40].

Protein digestion represents the first critical step in this process and differs markedly between animal-and plant-based foods. In general, animal-based proteins exhibit higher digestibility than plant-based proteins. Human studies indicate that approximately 85–95% of protein from sources such as egg, chicken, and dairy is digested and absorbed, compared with ~50–75% from legumes, including chickpeas, mung beans, and yellow peas. The lower digestibility of plant-based proteins is partly explained by the presence of anti-nutritional factors, such as dietary fiber and polyphenolic compounds, which can limit protein breakdown and amino acid absorption [40].

Following digestion, amino acid absorption and postprandial kinetics play a key role in determining systemic amino acid availability and the subsequent stimulation of MPS. The rate at which amino acids appear in circulation is strongly influenced by protein sources. Rapidly digested proteins, such as whey, induce a large but transient increase in plasma amino acids and leucine concentrations, whereas more slowly digested proteins, such as casein, result in a more moderate but sustained aminoacidemia due to gastric coagulation and delayed release. Plant-based proteins, such as soy, generally display intermediate kinetics but often elicit lower peak leucine concentrations compared with isonitrogenous doses of whey [18,25,39,40]. A large, pooled analysis of tracer studies (*n* = 602) showed that, over 5 h postprandially, ~57% of whey-, 45% of casein- and 65% of milk-derived phenylalanine appeared in the circulation, with older individuals exhibiting lower overall amino acid availability than younger adults [41]. These data underscore how both protein type and age modulate systemic amino acid availability and thereby the capacity to stimulate MPS.

Protein quality is determined by both the digestibility of a protein and its indispensable amino acid composition. The Protein Digestibility-Corrected Amino Acid Score (PDCAAS) has historically been used to evaluate protein quality, but it has important limitations, including truncation of values at 1.0 and reliance on fecal digestibility. To address these issues, the FAO proposed the Digestible Indispensable Amino Acid Score (DIAAS), which uses ileal digestibility for individual amino acids and does not truncate values > 1.0 [17,19]. According to FAO-supported analyses, high-quality animal-based proteins such as milk, whey, casein, egg and most meats typically have DIAAS values ≥ 1.0 (≥100), indicating that they meet or exceed requirements for all indispensable amino acids after digestion [17,19]. Many plant-based proteins have lower DIAAS values, with some legumes limited in sulphur amino acids (methionine + cysteine) and most cereals limited in lysine. However, certain plant isolates or concentrates—soy, potato, pea, canola—approach or reach DIAAS values comparable to animal-based proteins [18,19]. For a given protein source, the “limiting” amino acid is the indispensable amino acid present in the lowest proportion relative to requirements. Cereals (wheat, rice, maize) are typically lysine-limited, while legumes (soy, lentils, chickpeas) are often methionine-limited. Blending cereals with legumes, therefore, improves the overall amino acid score, yielding a composite profile closer to reference values and effectively increasing DIAAS [17,18,19].

This principle is particularly relevant for older adults shifting towards plant-based diets: combining lysine-poor cereals with methionine-poor legumes (e.g., pasta and beans, rice and lentils, wholegrain bread and hummus) can mitigate individual limitations and support adequate essential amino acid (EAA) exposure per meal [18]. A practical challenge in older adults is that plant-based foods generally provide less protein per unit energy than animal foods. To achieve a given protein and leucine dose (e.g., 30 g protein and 3 g leucine), considerably larger portions of many plant foods are required, increasing energy intake and gastric load. This may be problematic in older individuals with reduced appetite, early satiety, or cardiometabolic comorbidities requiring energy restriction [8,9,42]. High-protein plant foods (soy-based products, mycoprotein, seitan, certain legumes and nuts) and isolated plant-based proteins can partly overcome this limitation by providing more protein-dense options that fit within energy constraints [18,43,44]. Animal-based proteins (whey, casein, meat, eggs, fish) typically contain ~8–12% of protein as leucine, whereas many plant-based proteins (e.g., wheat, rice) have lower leucine density; soy and some novel plant-based proteins (corn, potato, pea isolates) are relatively leucine-rich [18,39,41,45]. Consequently, achieving the leucine threshold with plant-based proteins often requires larger absolute protein portions or the careful selection and blending of leucine-rich sources [18,39,45].

Beyond age-related declines in appetite, aging is accompanied by progressive impairments in gustatory and olfactory function that substantially influence energy intake and protein consumption in older adults. Age-associated losses in taste and smell are linked to reduced appetite, altered food preferences, and lower overall energy intake and key nutrients, including protein [46]. In particular, diminished sensitivity to umami selectively impairs the perception of savory, aroma-rich foods such as meat, reducing their palatability and consumption. Concurrently, olfactory sensitivity to savory food odors, including those characteristics of meat, declines disproportionately relative to other odor categories. Collectively, these sensory impairments are associated with adverse dietary patterns, characterized by reduced intake of protein-dense foods, especially meat [47,48,49]. In this context, plant-based protein sources may represent a viable alternative, as they typically exhibit milder aromas and, in many cases, softer textures than meat. These properties may be especially advantageous for older adults with compromised oral function, including chewing difficulties related to tooth loss or the use of dental prostheses.

**From a practical and translational perspective**, the application of protein quality metrics such as DIAAS requires consideration of the whole-diet context rather than isolated foods. In mixed meals, interactions between protein sources can improve overall amino acid availability, thereby reducing the relevance of single-protein DIAAS values [17,50,51,52]. Accordingly, dietary protein evaluation in free-living conditions should prioritize total protein intake and meal composition over individual food scores [8,51]. In addition, physiological factors—particularly in older adults, including impaired digestion, increased splanchnic extraction, and reduced anabolic sensitivity—further complicate the direct translation of protein quality metrics into functional outcomes [53,54].

An integrative framework may therefore be more informative for guiding dietary recommendations. First, achieving sufficient total daily protein intake remains the primary determinant of adequate amino acid availability [8,9]. Second, distributing protein intake evenly across meals, with each meal providing an adequate leucine stimulus, may help maximize postprandial MPS [25,55,56]. Third, protein quality considerations should be applied pragmatically: prioritizing leucine-rich and highly digestible protein sources when anabolic sensitivity is reduced, while leveraging amino acid complementarity (e.g., cereal–legume combinations) in predominantly plant-based diets [17,50,52,57]. Finally, in populations with elevated anabolic thresholds, such as older adults, the use of protein-dense foods, fortified products, or isolated protein sources may be necessary to reconcile protein requirements with energy constraints [9,58].

Collectively, these considerations suggest that leucine content, digestibility, and amino acid complementarity should not be viewed as competing priorities but rather as complementary components within a broader dietary strategy. Interpreting protein quality within the context of total diet, meal structure, and individual physiology provides a more clinically and nutritionally relevant framework than reliance on a single metric alone [17,50,51].

Table 1 describes the protein quality profile of common animal and plant foods: digestibility, absorption speed, digestible indispensable amino acid score (DIAAS), limiting indispensable amino acids, protein density and leucine content [17,50,52,59,60,61,62,63,64,65,66].

## 5. Acute Muscle Protein Synthetic Responses: Plant Versus Animal-Based Proteins

Acute tracer studies using stable isotope-labeled amino acid techniques are considered the standard for measuring postprandial myofibrillar protein synthesis (MPS) rates in vivo and for analyzing the molecular factors influencing the anabolic response to protein intake. The overall body of evidence suggests that animal-based proteins tend to elicit a greater acute MPS response than most plant-based proteins gram-for-gram, but this difference varies across studies, reflecting multiple interacting factors, including protein source composition, processing, and the research population and conditions. Indeed, Age, physiological context (rest versus post-resistance exercise), protein dosage, and whether protein is ingested as an isolate or in a whole-food matrix all influence these variations [39,45].

In young adults, Jason E. Tang and colleagues administered isonitrogenous doses (22 g) of whey hydrolysate, casein, and soy protein isolate under both resting and post-exercise conditions [45]. At rest, muscle protein synthesis (MPS) rates did not differ substantially between protein sources. However, following exercise, whey hydrolysate significantly stimulated MPS to a greater extent than both casein and soy protein isolate. This effect was attributed to its higher leucine content and rapid digestion kinetics, which promote an early and pronounced peak in plasma leucine concentrations [45]. Even more, Tang JE., Trommelen J. showed that ingestion of whey, casein or soy proteins after resistance exercise increases mixed or myofibrillar MPS, but whey typically produces the largest response, followed by casein, with soy showing a smaller effect at equal protein doses [39,45]. These findings were corroborated in a whole-food context by Kevin D. Wilkinson et al., who demonstrated that fluid skim milk ingestion after resistance exercise in young men resulted in greater net phenylalanine uptake—a proxy for MPS—compared to an isonitrogenous soy beverage [67]. This suggests that the anabolic advantage of dairy proteins extends beyond isolated protein sources [67]. However, more recent evidence challenges this concept. I. van der Heijden et al. reported that in trained individuals, a plant-based protein blend (pea, rice, and canola) stimulated post-exercise myofibrillar protein synthesis rates comparable to whey protein, despite a lower essential amino acid (EAA) content [68].

Under resting conditions, different results have been observed. P. J. M. Pinckaers et al. found no significant differences in MPS rates when young males consumed 30 g of wheat protein isolate, milk protein concentrate, or a 50:50 blend [69]. These findings suggest that even lower-quality plant-based proteins, such as wheat isolate, can provide sufficient leucine availability to surpass the mTORC1 activation threshold in young adults when consumed in adequate amounts at rest [69]. This observation was further supported by subsequent work showing that a 30 g plant-based protein blend (wheat, corn, and pea) stimulated MPS to a similar extent as 30 g of milk protein in resting young males [70]. Moreover, fortification strategies appear effective: the addition of leucine to plant-based blends (e.g., pea and canola) can elevate MPS responses to levels comparable to whey protein in both young men and women [71]. Taken together, these findings indicate that the anabolic advantage of animal-based proteins in young adults is most evident at lower doses and in the post-exercise state. This advantage is markedly attenuated when plant-based protein intake is increased, blended, or fortified to optimize amino acid composition.

In older individuals, the phenomenon of anabolic resistance elevates the leucine threshold required for mTORC1 activation (approximately 3–4 g per meal vs. 2–3 g in younger adults) and attenuates the muscle protein synthesis (MPS) response to a given protein dose [20,21]. This makes the comparison of protein sources particularly relevant in aging populations. Consistent with this, evidence in elderly men shows that whey protein isolate, at doses of ~20–40 g, robustly stimulates myofibrillar MPS both at rest and following exercise. In contrast, equivalent doses of soy protein isolate elicit smaller responses, largely due to their lower leucine content and a less pronounced or slower rise in postprandial aminoacidemia. However, when soy protein is consumed in higher quantities to match leucine delivery, MPS responses can approach those observed with whey protein [39,45]. These observations align with the findings of Y. Yang et al., who reported that 20 g of soy protein isolate stimulated myofibrillar MPS to a significantly lesser extent than an equivalent dose of whey protein in older men, both at rest and after exercise [72]. Although increasing the soy protein dose to 40 g partially attenuated this difference, it did not fully restore equivalence [72]. Gorissen et al. and colleagues demonstrated that increasing the dose of wheat protein hydrolysate to 60 g significantly elevated MPS above fasting levels in healthy older men, highlighting that plant-based proteins retain anabolic potential when consumed in sufficiently high quantities [73]. When considering whole-food meals, Philippe J. M. Pinckaers et al. compared postprandial MPS responses in older adults following an omnivorous meal versus an isocaloric and isonitrogenous vegan meal (36 g protein each) [70]. The omnivorous meal elicited a significantly greater increase in myofibrillar MPS, accompanied by higher peak leucinemia and greater postprandial amino acid availability. These findings suggest that the food matrix of plant-based meals may impose additional limitations beyond those observed in isolated protein studies, and that matching total protein content alone is insufficient to equalize anabolic responses when protein quality and digestibility differ [70].

Nevertheless, longer-term data provide a more nuanced perspective. A randomized controlled crossover trial found no changes in the daily integrated MPS rates between an isocaloric and isonitrogenous omnivorous diet and a well-balanced vegan diet in a randomized controlled crossover trial in physically active older adults. Moreover, the vegan diet was associated with improvements in blood lipid profiles, including reductions in LDL, HDL, and total cholesterol. These results indicate that a carefully planned vegan diet incorporating diverse plant-based protein sources can support daily MPS rates comparable to those achieved with omnivorous diets in older populations [74].

Overall, acute postprandial studies tend to demonstrate a modest advantage for animal-based proteins in older adults or indicate the need for compensatory strategies—such as higher protein doses or blending of plant sources—to achieve comparable MPS stimulation. However, longer-term investigations suggest that when total protein intake is sufficient and dietary protein sources are varied, plant-based diets can support equivalent daily muscle protein synthesis despite the presence of anabolic resistance.

**From a practical and translational perspective,** it has been shown that three complementary approaches are effective in reducing the anabolic difference between plant- and animal-based proteins. First, poorer leucine density and digestibility can be adjusted by increasing the absolute amount of plant-based protein, but at the cost of increased energy intake and volume, a significant limitation in older persons with decreased appetite. Second, in both young and older adults, leucine or EAA fortification of inadequate plant-based protein dosages raises anabolic efficiency without significantly increasing caloric intake by restoring MPS to levels reached with animal-based protein. Third, combining plant-based proteins with complementary amino acid profiles, legumes (sulfur amino acid-limited, lysine-rich) with cereals (lysine-limited, leucine-adequate), improves the overall indispensable amino acid score and raises DIAAS toward values similar to animal-based proteins, also producing a more persistent aminoacidemia [8,9,18,23].

In summary, animal-based proteins are more anabolically effective per gram; this difference is especially evident in older persons, after exercise in young people, and when plant-based proteins are eaten as complete foods rather than isolates. In clinical and geriatric settings, when intake is limited, this gap, which can be addressed through dosage optimization, leucine fortification, and strategic blending, represents a significant practical challenge.

## 6. Long-Term Effects of Plant Versus Animal-Based Protein on Muscle Mass and Function

Classical work with soy protein concentrates showed that well-processed soy can serve as the sole source of nitrogen and indispensable amino acids for long-term maintenance of protein nutritional status in young men, at intakes around 0.8 g/kg/day, as evidenced by neutral or slightly positive nitrogen balance and stable functional measures [43]. These findings, however, do not directly address optimal protein intakes and quality requirements in older, sarcopenic or immobilized populations. When compared at equal doses and within the context of progressive resistance training, soy and whey protein supplementation commonly lead to similar increases in lean body mass and strength. A meta-analysis of long-term studies found no significant difference between soy and animal (mostly dairy) protein on gains in lean mass or strength in response to resistance exercise [44]. As already described, individual trials from Wilkinson et al., comparing fluid milk with soy beverages after resistance training, reported greater early gains with milk, Hartman et al., demonstrated that long-term differences tended to be modest when total protein intake and training were adequate [67,75]. These findings reconcile apparent discrepancies between acute MPS studies and chronic training outcomes: small differences in the acute MPS response to individual meals may not translate into large differences in long-term muscle accretion when individuals habitually consume sufficient protein and energy and perform resistance training [25,39,44,45,75].

Observational studies in older adults suggest that total protein intake is more consistently associated with muscle mass than the animal-to-plant-based protein ratio. In the Guangzhou Nutrition and Health Study, higher intakes of total protein were associated with greater appendicular skeletal muscle mass in middle-aged and older Chinese adults, whereas the proportion of animal- versus plant-based protein showed weaker or no independent associations [42]. Similar findings are emerging from other cohorts, including recent data indicating that overall protein density and intake may outweigh protein source in determining muscle mass trajectories [76]. Nevertheless, some studies in Western populations report that animal-based protein, particularly from dairy and lean meat, is more strongly associated with muscle mass and strength than plant-based protein, likely reflecting higher leucine density and co-ingested nutrients (e.g., calcium, vitamin D). Conversely, higher plant-based protein intake often accompanies healthier overall dietary patterns and may be sufficient to support muscle maintenance if total intake and physical activity are adequate [2,8,9,18,42,76].

On the other hand, plant-based diets may be deficient in certain essential nutrients if not carefully planned or appropriately supplemented. Notably, such dietary patterns have been associated with lower vitamin B_12_ status and reduced creatine availability compared with omnivorous diets. Vitamin B_12_ and creatine are derived predominantly from animal-source foods; thus, reducing or eliminating these foods leads to decreased intake unless compensated through fortified foods or supplements [77,78]. Creatine is absent from plant foods in physiologically meaningful amounts, and although endogenous synthesis from amino acid precursors occurs, dietary intake from meat and fish contributes substantially to whole-body creatine stores. Accordingly, lower dietary creatine intake is associated with reduced plasma and skeletal muscle creatine concentrations, particularly in vegans and long-term vegetarians [78,79]. Furthermore, the complete elimination of meat and its replacement with refined plant-based proteins may confer a greater risk of vitamin B_12_ deficiency, with potential implications for long-term health [77,80]. Collectively, these observations underscore the need for careful nutritional planning of plant-based protein diets, including targeted supplementation where necessary.

## 7. Cardiometabolic Health, Environmental Sustainability and Protein Sources

Large prospective cohorts have consistently reported that higher plant-based protein intake is associated with lower all-cause and cardiovascular mortality. In pooled analyses of the Nurses’ Health Study and Health Professionals Follow-up Study, replacing 3% of energy from animal-based protein (particularly processed red meat) with plant-based protein was associated with substantially lower mortality risk [12]. Studies in other populations have confirmed inverse associations between plant-based protein and mortality, whereas animal-based protein—especially from processed meats—is often positively associated with cardiovascular outcomes [13,14]. A dose–response meta-analysis of prospective cohorts found that dietary plant-based protein intake was inversely associated with all-cause and cardiovascular mortality, while higher intakes of animal-based protein showed neutral or adverse associations depending on the source (red vs. white meat, processed vs. unprocessed) [14]. More recent analyses focusing on the plant-to-animal-based protein ratio highlight that higher ratios are associated with lower cardiovascular disease and coronary artery disease risk, particularly when combined with higher total protein density [15]. Animal agriculture, especially ruminant meat production, is a major contributor to greenhouse gas emissions, land use and freshwater consumption. Comparative life cycle assessments show that, per 100 g of protein, beef and lamb have far higher greenhouse gas emissions and land use than poultry, eggs and most plant-based protein sources [10,16]. Legumes and many cereals exhibit the lowest GHG emissions per 100 g of protein, although differences in nutrient density and amino acid profile must be considered [10,11,16]. The EAT–Lancet Commission and subsequent planetary health diet analyses therefore recommend dietary patterns that are predominantly plant-based, with modest amounts of animal-based foods, to align human health and environmental sustainability [10,11]. Recent work further suggests that plant-based protein strategies (including novel plant-based meat analogs) can markedly reduce environmental impacts, though careful attention to processing, nutrient density and affordability is required [10,11,16]. From a geriatric and clinical nutrition perspective, a key concern is whether shifting towards plant-based protein might compromise muscle health in vulnerable older adults. The available evidence suggests that this risk can be mitigated by ensuring: adequate total protein intake (≥1.0–1.2 g/kg/day in healthy older adults, higher in illness) [8,9]; sufficient leucine and EAA exposure per meal, respecting the higher per-meal threshold in older adults [2,20,21,25]; and appropriate use of high-quality plant-based proteins (e.g., soy, pea, potato, mycoprotein, blends) and complementary combinations of plant foods [17,18,19,43,44]. Within this framework, increasing the plant-to-animal-based protein ratio is compatible with preserving muscle mass and function and may confer additional cardiometabolic and environmental benefits [10,11,12,15,18,42,44,76].

## 8. Practical Implications and Future Perspectives

This review highlights several pragmatic messages for clinicians, dietitians and researchers. Animal-based proteins remain highly efficient tools for overcoming anabolic resistance because of their high DIAAS, leucine density and favorable digestion kinetics. They are particularly useful in very frail, catabolic or anorexic patients, and during early rehabilitation or post-hospitalization when energy and intake are limited [2,3,39,41,45]. Plant-based proteins, however, can also support muscle maintenance when specific conditions are met. Total daily protein intake should reach at least 1.0–1.2 g/kg/day, or higher where clinically indicated [8,9]. Protein intake should also be evenly distributed across meals—approximately 0.4 g/kg per meal, with at least 3 g of leucine per meal in older adults [2,20,21,25]. In addition, emphasis should be placed on high-quality plant proteins and complementary protein blends to ensure an adequate amino acid profile [17,18,19,43,44]. Soy is often considered a model plant-based protein in this context. It provides a complete amino acid profile, is highly versatile for food development (e.g., meat analogs and beverages), and offers additional nutritional benefits such as fiber and essential minerals. Moreover, soy is relatively affordable and has been extensively studied for its potential health benefits. Well-processed soy concentrates and isolates have demonstrated capacity to maintain nitrogen balance and provide all indispensable amino acids in adults, and experimental and clinical studies show that soy-based diets or supplements can maintain lean mass and strength when total protein and energy are adequate [18,43,44,72]. Blending and fortification strategies can meaningfully improve the anabolic quality of plant-based diets. Combining cereal and legume proteins enhances amino acid profiles and increases DIAAS, while targeted leucine fortification—or co-ingestion of leucine-rich plant protein sources such as soy, corn, or potato protein isolates—can help older adults reach the leucine threshold without excessive energy intake [17,18,19,20,21].

However, dietary strategies alone are not sufficient. Resistance exercise remains indispensable, as it synergizes with protein intake to counteract anabolic resistance. Consuming protein-dense meals before or after exercise further enhances the utilization of dietary amino acids for muscle protein synthesis (MPS) in both young and older adults [1,2,3,25,39,45]. In practice, even the highest-quality protein source is unlikely to preserve muscle mass in the absence of adequate mechanical loading.

Context-specific considerations are also critical. In highly catabolic conditions—such as acute hospitalization, bed rest, or cachexia—where food intake is often limited and the anabolic window is constrained, animal-based proteins or fortified, high-quality plant protein isolates may be preferable to maximize anabolic efficiency per unit of energy and volume [3,8,9,18,39,41,45]. By contrast, in stable, community-dwelling older adults with sufficient energy intake, plant-forward dietary patterns that meet overall protein and leucine targets appear adequate for maintaining muscle mass, while also offering cardiometabolic and environmental benefits [2,8,9,10,11,12,15,18,42,76].

Reconciling the tension between anabolic efficiency and sustainability requires a pragmatic, multi-level framework that integrates protein quality with whole-diet context and individual physiology. At the meal level, maximizing postprandial muscle protein synthesis (MPS) remains critical, particularly in older adults and other populations exhibiting anabolic resistance [1,2]. This can be achieved by ensuring sufficient leucine availability through the selection of highly digestible, leucine-rich protein sources or, in predominantly plant-based meals, through strategies such as increased protein dosing, amino acid fortification, and the use of complementary protein blends (e.g., cereal–legume combinations) to improve indispensable amino acid profiles [17,18,19,20,21,39,45]. At the daily intake level, total protein consumption emerges as the primary determinant of net anabolic outcomes, with evidence indicating that adequate intake and balanced distribution across meals can largely compensate for differences in protein quality observed in acute studies. At the dietary pattern level, increasing the plant-to-animal-based protein ratio aligns with cardiometabolic and environmental health objectives, provided that nutritional adequacy is maintained through careful planning, including the use of protein-dense plant foods, fortified products, and supplementation where necessary (e.g., vitamin B_12_, potentially creatine) [10,12,15,16,77,78,79]. Importantly, long-term intervention and observational data suggest that, despite lower per-meal anabolic efficiency, well-designed plant-based diets can support comparable muscle mass and functional outcomes when total protein intake and physical activity are sufficient [25,39,44,67,74,75]. Future research should therefore move beyond reductionist protein quality metrics toward integrative models that incorporate digestion kinetics, food matrix effects, and inter-individual variability, while also advancing technological innovations such as improved plant-based protein processing, targeted amino acid fortification, and novel protein sources. Collectively, this framework supports a shift toward “precision protein nutrition,” in which protein recommendations are tailored to physiological needs while simultaneously addressing sustainability constraints. Table 2 provides a multi-level, practical framework for strategic protein use, integrating muscle maintenance with sustainability considerations.

## 9. Conclusions

In conclusion, current evidence does not support a simplistic dichotomy in which animal-based protein is “good for muscle but bad for health and the planet” and plant-based protein is the opposite. Instead, the data point to a nuanced picture. Per gram, animal-based proteins—particularly dairy and meat—are more anabolic, more protein-dense and often more practical for overcoming anabolic resistance in older or immobilized individuals [2,3,18,39,41,45,67,72,75]. However, plant-based proteins can effectively sustain muscle mass and function when total intake, leucine exposure and amino acid complementarity are optimized, especially in combination with resistance exercise [18,21,23,25,39,43,44,45,75]. At the population level, shifting the plant-to-animal-based protein ratio upwards appears desirable for cardiometabolic health and environmental sustainability, and is compatible with preserving muscle health in most older adults [10,11,12,14,15,16,42,44,76]. Protein intake should be age-stratified, with mixed protein sources recommended overall. In young, healthy adults, a higher proportion of plant-based proteins is associated with improved cardiovascular risk profiles, whereas in older adults, a greater contribution of animal-based proteins—due to their higher essential amino acid content and digestibility—is advisable to support muscle protein synthesis and mitigate sarcopenia. Within such a framework, plant-based proteins are not a universal “key”, but they are essential components of a sustainable toolkit to support both muscle health and planetary health—provided we respect the basic rules of protein biology: enough, well-distributed, leucine-rich and combined with movement. Table 3 summarizes the comparative roles of animal-and plant-based proteins across intake, anabolic properties, health outcomes, and sustainability, highlighting key practical considerations for their use.

Importantly, several limitations in the current evidence base should be acknowledged. Much of the available literature remains heterogeneous in study design, population characteristics, protein sources, and outcome measures, with a relative paucity of long-term, well-controlled trials directly comparing plant- and animal-based protein strategies in clinically relevant populations. As a result, the current evidence base is insufficient to define precise plant-to-animal protein ratios across specific age groups or clinical contexts. At present, only pragmatic, context-dependent guidance can be offered. Furthermore, the literature has tended to emphasize anabolic outcomes in isolation, often without adequately integrating cardiometabolic health, functional outcomes, and environmental considerations within the same analytical framework. This limits the ability to formulate fully evidence-based, multidimensional recommendations. Taken together, these gaps reinforce the need for a more integrative research agenda and support a cautious, individualized approach to protein recommendations across the lifespan. Future research priorities include: defining optimal plant-based protein combinations and fortification strategies for sarcopenic and multimorbid patients; integrating high-quality plant-based proteins into clinical pathways for prehabilitation, bed rest and post-acute rehabilitation; refining protein recommendations that explicitly incorporate protein quality (e.g., DIAAS) and leucine density; and quantifying trade-offs and synergies between muscle health, cardiometabolic risk and environmental impacts in different protein transition scenarios [10,11,12,16,18].

## Figures and Tables

**Table 1:** Protein quality profile of common animal and plant foods (DIAAS, digestibility, limiting amino acids, protein density and leucine content).

Protein Source	Origin	Approx. Digestibility	Relative Absorption Speed	Typical DIAAS	Limiting Indispensable AA	Protein Density (g/100 Food g %)	Approx. Leucine Content (g/100 g Protein %)	Note
Whey protein isolate (powder)	Animal	95–99%	Fast	≈1.09	None	≈85–90	≈11–12	Very high-quality, leucine-rich “fast” protein.
Casein/caseinate (powder)	Animal	95–98%	Slow	≈1.2–1.3	None	≈85–90	≈8–9	“Slow” protein; sustained AA and leucine release.
Cow’s milk (whole/skim)	Animal	95–97%	Mixed (fast + slow)	≈1.10–1.18	None	≈3–4	≈9–10	80% casein, 20% whey: high leucine fraction.
Yogurt/fermented milk	Animal	95–97%	Intermediate	≈1.0–1.1	None	≈5–10	≈9–10	Similar AA profile to milk; density depends on style (regular vs. Greek).
Egg (whole, cooked)	Animal	95–98%	Intermediate	≈1.10–1.13	None	≈12–13	≈8–9	Classical reference protein; good leucine density.
Lean meat (beef, pork, lamb)	Animal	90–97%	Intermediate–slow	≈1.10–1.20	None	≈20–25	≈7–8	High-quality protein; leucine usually ~7–8% of protein.
Poultry (chicken, turkey)	Animal	90–97%	Intermediate	≈1.05–1.10	None	≈22–24	≈8–9	Skinless breasts are particularly leucine dense.
Fish (white fish, tuna)	Animal	90–97%	Intermediate	≈1.0–1.1	None	≈20–24	≈8–9	Easily digested, good leucine fraction.
Soy protein isolate	Plant	90–97%	Intermediate	≈0.90	Met + Cys	≈80–90	≈7.5–8.5	Most “animal-like” plant-based protein; good leucine, sulfur AAs limiting.
Tofu/tempeh (cooked)	Plant	85–95%	Intermediate –slow	≈0.90–0.97	Met + Cys	≈8–12 (tofu), 18–20 (tempeh)	≈7.5–8.5	Same AA pattern as soy; density depends on water content.
Pea protein isolate (powder)	Plant	90–97%	Intermediate –fast	≈0.82–1.00	Met + Cys	≈75–85	≈7.5–8.5	Good-quality plant-based protein; leucine moderate–high.
Whole lentils/beans/chickpeas (cooked)	Plant	80–90%	Slow	≈0.55–0.85	Met + Cys	≈7–9	≈7–8	Typical legume pattern: leucine adequate, sulfur AAs limiting.
Whole grains (wheat, oats, rice; cooked)	Plant	70–90%	Slow	≈0.40–0.60	Lysine	≈2–5	≈6–7	Leucine is generally sufficient relative to needs; lysine is limiting.
Nuts and seeds (almonds, peanuts, etc.)	Plant	70–85%	Slow	≈0.40–0.45	Lysine	≈15–25	≈7–9	Energy-dense; moderate protein and leucine density.

Abbreviations; EAA, essential amino acid(s); AA, amino acid(s); DIAAS, Digestible Indispensable Amino Acid Score; MET, methionine; CYS, cysteine. Leucine content in 100 g of food can be calculated as follows: protein density (g/100 g food%) × leucine content (g/100 g protein%)/100.

**Table 2:** Practical framework for protein use in daily practice.

Anabolic Efficiency ↔ Sustainability A Multi-Level Framework for Protein Nutrition	
	**Moderators**
**1. MEAL—LEVEL** Acute MPS optimization → Maximizes anabolic efficiency per meal Achieve leucine threshold − ~2–3 g young adults − ~3–4 g older adults Prioritize digestible, leucine-rich proteins Even distribution across meals: − ~20–25 g/meal in young adults − ~30–40 g/meal in older adults Plant strategies: − Protein blending (cereal + legume) − Protein isolates (soy, pea, potato) − Leucine/EAA fortification − Increase dose when using plant-based proteins	Age (anabolic resistance) Physical activity Appetite and energy intake Digestive efficiency Nutrient status (e.g., vitamin B_12_, creatine)
**↓**	
**2. DAILY INTAKE LEVEL** Total Protein Sufficiency → Compensates for lower protein quality Total protein intake: − ≥1.0–1.2 g/kg/day in older adults − ≥1.2–1.5 g/kg/day in acute or chronic illness, rehabilitation and sarcopenia Increase diversity of protein sources Use fortified/isolated proteins if needed (insufficient intake)	
**↓**	
**3. DIETARY PATTERN LEVEL** Sustainability Integration → Prioritizes sustainability and long-term health Increase plant-to-animal-based protein ratio Limit processed and red meat Emphasize legumes, whole grains, novel proteins Reserve animal-based proteins for aging, clinical states, post-exercise (Strategic use) Supplementing vitamin B_12_ and creatine on plant-forward diets	
**↓**	
**4. LONG-TERM OUTCOMES** When intake and training are adequate Muscle mass and function: comparable across protein sources Cardiometabolic health: higher plant-based protein → lower CVD risk Environmental impact: reduced GHG emissions, land and water use	
**Each level builds on the one above.** **Moderators (right) apply across all levels.**	

A multi-level practical framework illustrating how the tension between anabolic efficiency and sustainability in protein nutrition can be resolved. At the meal level, strategies focus on optimizing postprandial muscle protein synthesis through adequate leucine intake and protein quality. At the daily level, sufficient total protein intake and distribution compensate for differences in protein sources. At the dietary pattern level, increasing the proportion of plant-based proteins supports cardiometabolic health and environmental sustainability. Long-term outcomes indicate that, when total protein intake and physical activity are adequate, plant- and animal-based diets can support comparable muscle mass and function. Age-stratified protein intake should be considered for optimal muscle mass and cardiovascular health. Abbreviations: MPS, muscle protein synthesis; EAA, essential amino acid(s); CVD, cardiovascular disease; GHG, greenhouse gas.

**Table 3:** Summary: animal- based proteins versus plant-based proteins.

**Domain**	**Animal-Based Protein**	**Plant-Based Protein**
**Recommendation for** **protein intake**	Supports achievement of recommended intakes (≥1.0–1.2 g/kg/day; ≥1.2–1.5 g/kg/day in illness) with lower food volume	Can meet recommended intakes when total protein intake is adequate and meals are appropriately planned
**Per-meal protein** **distribution**	Facilitates achievement of ~0.4 g/kg/meal and sufficient leucine content (~2.5–3 g/meal)	Comparable per-meal targets apply; may require higher protein doses, protein blending, or leucine fortification
**Protein quality** **and** **digestibility**	Generally high digestibility and favorable indispensable amino acid profile	Lower digestibility and limiting amino acids; quality improves with complementary protein sources and fortification
**Muscle protein** **synthesis**	Effectively stimulates postprandial muscle protein synthesis at moderate doses	Comparable stimulation achievable with higher doses and/or leucine or EAA enrichment
**Muscle mass and function**	Effective for maintenance of muscle mass and function	Similar long-term outcomes when total intake and resistance exercise are sufficient
**Cardiometabolic** **health**	Health effects depend on protein source and overall dietary pattern	Diets emphasizing plant-based protein sources are associated with favorable cardiometabolic outcomes
**Environmental** **sustainability**	Higher environmental footprint	Lower environmental footprint
**Clinical and practical** **application**	Particularly useful in frail, sarcopenic, or acutely ill individuals with low appetite or energy intake	Appropriate for older adults, provided protein quantity, amino acid adequacy, and physical activity are ensured

Abbreviations; EAA, essential amino acid(s).

## Data Availability

No new data was generated for this study. All information analyzed is contained within the published literature.

## Abbreviations

The following abbreviations are used in this manuscript:

MPS	Muscle Protein Synthesis
EAA	Essential Amino Acid
mTORC1	Mammalian target of Rapamycin Complex 1
PROT-AGE	Protein- Aging Study Group
ESPEN	European Society for Clinical Nutrition and Metabolism
AMPK	Adenosine 5′-monophosphate -activated protein kinase
IGF-1	Insulin- like Growth Factor 1
IRS-1	Insulin Receptor Substrate-1
PI3K	Phosphoinositide 3-Kinase
p-Akt	Phosphorylated Protein Kinase B
FOXO	FOXO, forkhead box O
S6K1	Ribosomal Protein S6 Kinase 1
4E-BP1	Eukaryotic Initiation Factor 4E-Binding Protein 1
MURF1	Muscle RING-Finger Protein-1
MAFbx	Muscle Atrophy F-Box
UPS	Ubiquitin–Proteasome System
FAO	Food and Agriculture Organization
PDCAAS	Protein Digestibility-Corrected Amino Acid Score
DIAAS	Digestible Indispensable Amino Acid Score
MET	Methionine
CYS	Cysteine
LDL	Low-Density Lipoprotein
HDL	High-Density Lipoprotein
EAT	EAT Lancet Diet or Planetary Health Diet
CVD	Cardiovascular Disease
GHG	Greenhouse Gas

## References

[1] Paulussen K.J.M., McKenna C.F., Beals J.W., Wilund K.R., Salvador A.F., Burd N.A. (2021). Anabolic Resistance of Muscle Protein Turnover Comes in Various Shapes and Sizes. Front. Nutr..

[2] Moore D.R. (2014). Keeping Older Muscle “Young” through Dietary Protein and Physical Activity. Adv. Nutr..

[3] Biolo G., Pišot R., Mazzucco S., Di Girolamo F.G., Situlin R., Lazzer S., Grassi B., Reggiani C., Passaro A., Rittweger J. (2017). Anabolic resistance assessed by oral stable isotope ingestion following bed rest in young and older adult volunteers: Relationships with changes in muscle mass. Clin. Nutr..

[4] Biolo G., Cederholm T., Muscaritoli M. (2014). Muscle contractile and metabolic dysfunction is a common feature of sarcopenia of aging and chronic diseases: From sarcopenic obesity to cachexia. Clin. Nutr..

[5] Hoffer L.J., Bistrian B.R., Young V.R., Blackburn G.L., Matthews D.E. (1984). Metabolic effects of very low calorie weight reduction diets. J. Clin. Investig..

[6] Biolo G., Ciocchi B., Lebenstedt M., Barazzoni R., Zanetti M., Platen P., Heer M., Guarnieri G. (2004). Short-term bed rest impairs amino acid-induced protein anabolism in humans. J. Physiol..

[7] Campbell W.W., Deutz N.E.P., Volpi E., Apovian C.M. (2023). Nutritional Interventions: Dietary Protein Needs and Influences on Skeletal Muscle of Older Adults. J. Gerontol. A Biol. Sci. Med. Sci..

[8] Bauer J., Biolo G., Cederholm T., Cesari M., Cruz-Jentoft A.J., Morley J.E., Phillips S., Sieber C., Stehle P., Teta D. (2013). Evidence-Based Recommendations for Optimal Dietary Protein Intake in Older People: A Position Paper From the PROT-AGE Study Group. J. Am. Med. Dir. Assoc..

[9] Deutz N.E.P., Bauer J.M., Barazzoni R., Biolo G., Boirie Y., Bosy-Westphal A., Cederholm T., Cruz-Jentoft A., Krznariç Z., Nair K.S. (2014). Protein intake and exercise for optimal muscle function with aging: Recommendations from the ESPEN Expert Group. Clin. Nutr..

[10] Tilman D., Clark M. (2014). Global diets link environmental sustainability and human health. Nature.

[11] Willett W., Rockström J., Loken B., Springmann M., Lang T., Vermeulen S., Garnett T., Tilman D., DeClerck F., Wood A. (2019). Food in the Anthropocene: The EAT-Lancet Commission on healthy diets from sustainable food systems. Lancet.

[12] Song M., Fung T.T., Hu F.B., Willett W.C., Longo V.D., Chan A.T., Giovannucci E.L. (2016). Association of Animal and Plant Protein Intake with All-Cause and Cause-Specific Mortality. JAMA Intern. Med..

[13] Huang J., Liao L.M., Weinstein S.J., Sinha R., Graubard B.I., Albanes D. (2020). Association Between Plant and Animal Protein Intake and Overall and Cause-Specific Mortality. JAMA Intern. Med..

[14] Naghshi S., Sadeghi O., Willett W.C., Esmaillzadeh A. (2020). Dietary intake of total, animal, and plant proteins and risk of all cause, cardiovascular, and cancer mortality: Systematic review and dose-response meta-analysis of prospective cohort studies. BMJ.

[15] Glenn A.J., Wang F., Tessier A.-J., Manson J.E., Rimm E.B., Mukamal K.J., Sun Q., Willett W.C., Rexrode K.M., Jenkins D.J. (2024). Dietary plant-to-animal protein ratio and risk of cardiovascular disease in 3 prospective cohorts. Am. J. Clin. Nutr..

[16] Merlo M., Hennessy T., Buckley C., O’Mahony J. (2024). A comparison of animal and plant-based proteins from an economic, environmental, and nutritional perspective in the Republic of Ireland. Agric. Syst..

[17] FAO (2013). Dietary Protein Quality Evaluation in Human Nutrition. https://openknowledge.fao.org/handle/20.500.14283/i3124e.

[18] van Vliet S., Burd N.A., van Loon L.J.C. (2015). The Skeletal Muscle Anabolic Response to Plant- versus Animal-Based Protein Consumption. J. Nutr..

[19] Xipsiti M. (2024). Protein quality evaluation: FAO perspective. Front. Nutr..

[20] Katsanos C.S., Kobayashi H., Sheffield-Moore M., Aarsland A., Wolfe R.R. (2006). A high proportion of leucine is required for optimal stimulation of the rate of muscle protein synthesis by essential amino acids in the elderly. Am. J. Physiol. Endocrinol. Metab..

[21] Zaromskyte G., Prokopidis K., Ioannidis T., Tipton K.D., Witard O.C. (2021). Evaluating the Leucine Trigger Hypothesis to Explain the Post-prandial Regulation of Muscle Protein Synthesis in Young and Older Adults: A Systematic Review. Front. Nutr..

[22] Casperson S.L., Sheffield-Moore M., Hewlings S.J., Paddon-Jones D. (2012). Leucine supplementation chronically improves muscle protein synthesis in older adults consuming the RDA for protein. Clin. Nutr..

[23] Churchward-Venne T.A., Burd N.A., Mitchell C.J., West D.W.D., Philp A., Marcotte G.R., Baker S.K., Baar K., Phillips S.M. (2012). Supplementation of a suboptimal protein dose with leucine or essential amino acids: Effects on myofibrillar protein synthesis at rest and following resistance exercise in men. J. Physiol..

[24] Burd N.A., West D.W.D., Moore D.R., Atherton P.J., Staples A.W., Prior T., Tang J.E., Rennie M.J., Baker S.K., Phillips S.M. (2011). Enhanced amino acid sensitivity of myofibrillar protein synthesis persists for up to 24 h after resistance exercise in young men. J. Nutr..

[25] Moore D.R., Robinson M.J., Fry J.L., Tang J.E., Glover E.I., Wilkinson S.B., Prior T., Tarnopolsky M.A., Phillips S.M. (2009). Ingested protein dose response of muscle and albumin protein synthesis after resistance exercise in young men. Am. J. Clin. Nutr..

[26] Atherton P.J., Smith K. (2012). Muscle protein synthesis in response to nutrition and exercise. J. Physiol..

[27] Biolo G., Tipton K.D., Klein S., Wolfe R.R. (1997). An abundant supply of amino acids enhances the metabolic effect of exercise on muscle protein. Am. J. Physiol..

[28] Biolo G., Williams B.D., Fleming R.Y., Wolfe R.R. (1999). Insulin action on muscle protein kinetics and amino acid transport during recovery after resistance exercise. Diabetes.

[29] Koopman R., Beelen M., Stellingwerff T., Pennings B., Saris W.H.M., Kies A.K., Kuipers H., van Loon L.J.C. (2007). Coingestion of carbohydrate with protein does not further augment postexercise muscle protein synthesis. Am. J. Physiol. Endocrinol. Metab..

[30] Groen B.B.L., Horstman A.M.H., Hamer H.M., de Haan M., van Kranenburg J., Bierau J., Poeze M., Wodzig W.K.W.H., Rasmussen B.B., van Loon L.J.C. (2016). Increasing Insulin Availability Does Not Augment Postprandial Muscle Protein Synthesis Rates in Healthy Young and Older Men. J. Clin. Endocrinol. Metab..

[31] Di Donato D.M., West D.W.D., Churchward-Venne T.A., Breen L., Baker S.K., Phillips S.M. (2014). Influence of aerobic exercise intensity on myofibrillar and mitochondrial protein synthesis in young men during early and late postexercise recovery. Am. J. Physiol. Endocrinol. Metab..

[32] Sjøberg K.A., Frøsig C., Kjøbsted R., Sylow L., Kleinert M., Betik A.C., Shaw C.S., Kiens B., Wojtaszewski J.F.P., Rattigan S. (2017). Exercise Increases Human Skeletal Muscle Insulin Sensitivity via Coordinated Increases in Microvascular Perfusion and Molecular Signaling. Diabetes.

[33] Areta J.L., Burke L.M., Ross M.L., Camera D.M., West D.W.D., Broad E.M., Jeacocke N.A., Moore D.R., Stellingwerff T., Phillips S.M. (2013). Timing and distribution of protein ingestion during prolonged recovery from resistance exercise alters myofibrillar protein synthesis. J. Physiol..

[34] Fyfe J.J., Bishop D.J., Stepto N.K. (2014). Interference between concurrent resistance and endurance exercise: Molecular bases and the role of individual training variables. Sports Med..

[35] Donges C.E., Burd N.A., Duffield R., Smith G.C., West D.W.D., Short M.J., Mackenzie R., Plank L.D., Shepherd P.R., Phillips S.M. (2012). Concurrent resistance and aerobic exercise stimulates both myofibrillar and mitochondrial protein synthesis in sedentary middle-aged men. J. Appl. Physiol..

[36] Ely I.A., Phillips B.E., Smith K., Wilkinson D.J., Piasecki M., Breen L., Larsen M.S., Atherton P.J. (2023). A focus on leucine in the nutritional regulation of human skeletal muscle metabolism in ageing, exercise and unloading states. Clin. Nutr..

[37] Kitajima Y., Yoshioka K., Suzuki N. (2020). The ubiquitin-proteasome system in regulation of the skeletal muscle homeostasis and atrophy: From basic science to disorders. J. Physiol. Sci..

[38] Wackerhage H., Schoenfeld B.J., Hamilton D.L., Lehti M., Hulmi J.J. (2019). Stimuli and sensors that initiate skeletal muscle hypertrophy following resistance exercise. J. Appl. Physiol..

[39] Trommelen J., Betz M.W., van Loon L.J.C. (2019). The Muscle Protein Synthetic Response to Meal Ingestion Following Resistance-Type Exercise. Sports Med..

[40] Pinckaers P.J.M., Trommelen J., Snijders T., van Loon L.J.C. (2021). The Anabolic Response to Plant-Based Protein Ingestion. Sports Med..

[41] Gorissen S.H.M., Trommelen J., Kouw I.W.K., Holwerda A.M., Pennings B., Groen B.B.L., Wall B.T., Churchward-Venne T.A., Horstman A.M.H., Koopman R. (2020). Protein Type, Protein Dose, and Age Modulate Dietary Protein Digestion and Phenylalanine Absorption Kinetics and Plasma Phenylalanine Availability in Humans. J. Nutr..

[42] Li C.-Y., Fang A.-P., Ma W.-J., Wu S.-L., Li C.-L., Chen Y.-M., Zhu H.-L. (2019). Amount Rather than Animal vs Plant Protein Intake Is Associated with Skeletal Muscle Mass in Community-Dwelling Middle-Aged and Older Chinese Adults: Results from the Guangzhou Nutrition and Health Study. J. Acad. Nutr. Diet..

[43] Istfan N., Murray E., Janghorbani M., Evans W.J., Young V.R. (1983). The nutritional value of a soy protein concentrate (STAPRO-3200) for long-term protein nutritional maintenance in young men. J. Nutr..

[44] Messina M., Lynch H., Dickinson J.M., Reed K.E. (2018). No Difference Between the Effects of Supplementing with Soy Protein Versus Animal Protein on Gains in Muscle Mass and Strength in Response to Resistance Exercise. Int. J. Sport Nutr. Exerc. Metab..

[45] Tang J.E., Moore D.R., Kujbida G.W., Tarnopolsky M.A., Phillips S.M. (2009). Ingestion of whey hydrolysate, casein, or soy protein isolate: Effects on mixed muscle protein synthesis at rest and following resistance exercise in young men. J. Appl. Physiol..

[46] Honnens De Lichtenberg Broge E., Wendin K., Rasmussen M.A., Bredie W.L.P. (2021). Changes in perception and liking for everyday food odors among older adults. Food Qual. Prefer..

[47] Fluitman K.S., Hesp A.C., Kaihatu R.F., Nieuwdorp M., Keijser B.J.F., Ijzerman R.G., Visser M. (2021). Poor Taste and Smell Are Associated with Poor Appetite, Macronutrient Intake, and Dietary Quality but Not with Undernutrition in Older Adults. J. Nutr..

[48] Schiffman S., Graham B.G. (2000). Taste and smell perception affect appetite and immunity in the elderly. Eur. J. Clin. Nutr..

[49] Sergi G., Bano G., Pizzato S., Veronese N., Manzato E. (2017). Taste loss in the elderly: Possible implications for dietary habits. Crit. Rev. Food Sci. Nutr..

[50] Gorissen S.H.M., Crombag J.J.R., Senden J.M.G., Waterval W.A.H., Bierau J., Verdijk L.B., van Loon L.J.C. (2018). Protein content and amino acid composition of commercially available plant-based protein isolates. Amino Acids.

[51] Wolfe R.R. (2017). Branched-chain amino acids and muscle protein synthesis in humans: Myth or reality?. J. Int. Soc. Sports Nutr..

[52] Mathai J.K., Liu Y., Stein H.H. (2017). Values for digestible indispensable amino acid scores (DIAAS) for some dairy and plant proteins may better describe protein quality than values calculated using the concept for protein digestibility-corrected amino acid scores (PDCAAS). Br. J. Nutr..

[53] Moore D.R., Churchward-Venne T.A., Witard O., Breen L., Burd N.A., Tipton K.D., Phillips S.M. (2015). Protein ingestion to stimulate myofibrillar protein synthesis requires greater relative protein intakes in healthy older versus younger men. J. Gerontol. A Biol. Sci. Med. Sci..

[54] Breen L., Phillips S.M. (2011). Skeletal muscle protein metabolism in the elderly: Interventions to counteract the ‘anabolic resistance’ of ageing. Nutr. Metab..

[55] Witard O.C., Jackman S.R., Breen L., Smith K., Selby A., Tipton K.D. (2014). Myofibrillar muscle protein synthesis rates subsequent to a meal in response to increasing doses of whey protein at rest and after resistance exercise. Am. J. Clin. Nutr..

[56] Mamerow M.M., Mettler J.A., English K.L., Casperson S.L., Arentson-Lantz E., Sheffield-Moore M., Layman D.K., Paddon-Jones D. (2014). Dietary protein distribution positively influences 24-h muscle protein synthesis in healthy adults. J. Nutr..

[57] Young V.R., Pellett P.L. (1994). Plant proteins in relation to human protein and amino acid nutrition. Am. J. Clin. Nutr..

[58] Houston D.K., Nicklas B.J., Ding J., Harris T.B., Tylavsky F.A., Newman A.B., Lee J.S., Sahyoun N.R., Visser M., Kritchevsky S.B. (2008). Dietary protein intake is associated with lean mass change in older, community-dwelling adults: The Health, Aging, and Body Composition (Health ABC) Study. Am. J. Clin. Nutr..

[59] Boye J., Wijesinha-Bettoni R., Burlingame B. (2012). Protein quality evaluation twenty years after the introduction of the protein digestibility corrected amino acid score method. Br. J. Nutr..

[60] Herreman L., Nommensen P., Pennings B., Laus M.C. (2020). Comprehensive overview of the quality of plant- And animal-sourced proteins based on the digestible indispensable amino acid score. Food Sci. Nutr..

[61] Rutherfurd S.M., Fanning A.C., Miller B.J., Moughan P.J. (2015). Protein digestibility-corrected amino acid scores and digestible indispensable amino acid scores differentially describe protein quality in growing male rats. J. Nutr..

[62] Fanelli N.S., Bailey H.M., Guardiola L.V., Stein H.H. (2021). Values for Digestible Indispensable Amino Acid Score (DIAAS) Determined in Pigs Are Greater for Milk Than for Breakfast Cereals, but DIAAS Values for Individual Ingredients Are Additive in Combined Meals. J. Nutr..

[63] Cervantes-Pahm S.K., Liu Y., Stein H.H. (2014). Digestible indispensable amino acid score and digestible amino acids in eight cereal grains. Br. J. Nutr..

[64] Hall C., Hillen C., Garden Robinson J. (2017). Composition, Nutritional Value, and Health Benefits of Pulses. Cereal Chem..

[65] Ajomiwe N., Boland M., Phongthai S., Bagiyal M., Singh J., Kaur L. (2024). Protein Nutrition: Understanding Structure, Digestibility, and Bioavailability for Optimal Health. Foods.

[66] Boirie Y., Dangin M., Gachon P., Vasson M.-P., Maubois J.-L., Beaufrère B. (1997). Slow and fast dietary proteins differently modulate postprandial protein accretion. Proc. Natl. Acad. Sci. USA.

[67] Wilkinson S.B., Tarnopolsky M.A., MacDonald M.J., Macdonald J.R., Armstrong D., Phillips S.M. (2007). Consumption of fluid skim milk promotes greater muscle protein accretion after resistance exercise than does consumption of an isonitrogenous and isoenergetic soy-protein beverage. Am. J. Clin. Nutr..

[68] Van Der Heijden I., Monteyne A.J., West S., Morton J.P., Langan-Evans C., Hearris M.A., Abdelrahman D.R., Murton A.J., Stephens F.B., Wall B.T. (2024). Plant Protein Blend Ingestion Stimulates Postexercise Myofibrillar Protein Synthesis Rates Equivalently to Whey in Resistance-Trained Adults. Med. Sci. Sports Exerc..

[69] Pinckaers P.J.M., Kouw I.W.K., Hendriks F.K., van Kranenburg J.M., de Groot L.C., Verdijk L.B., Snijders T., van Loon L.J. (2021). No differences in muscle protein synthesis rates following ingestion of wheat protein, milk protein, and their protein blend in healthy, young males. Br. J. Nutr..

[70] Pinckaers P.J.M., Kouw I.W.K., Gorissen S.H.M., Houben L.H.P., Senden J.M., Wodzig W.K.H.W., de Groot L.C.P.G.M., Verdijk L.B., Snijders T., van Loon L.J.C. (2023). The Muscle Protein Synthetic Response to the Ingestion of a Plant-based Protein Blend Does Not Differ from an Equivalent Amount of Milk Protein in Healthy Young Males. J. Nutr..

[71] Lim C., Janssen T.A.H., Currier B.S., Paramanantharajah N., McKendry J., Abou Sawan S.A., Phillips S.M. (2024). Muscle Protein Synthesis in Response to Plant-Based Protein Isolates with and Without Added Leucine Versus Whey Protein in Young Men and Women. Curr. Dev. Nutr..

[72] Yang Y., Churchward-Venne T.A., Burd N.A., Breen L., Tarnopolsky M.A., Phillips S.M. (2012). Myofibrillar protein synthesis following ingestion of soy protein isolate at rest and after resistance exercise in elderly men. Nutr. Metab..

[73] Gorissen S.H., Horstman A.M., Franssen R., Crombag J.J., Langer H., Bierau J., Respondek F., van Loon L.J. (2016). Ingestion of Wheat Protein Increases In Vivo Muscle Protein Synthesis Rates in Healthy Older Men in a Randomized Trial. J. Nutr..

[74] Domić J., Pinckaers P.J., Grootswagers P., Siebelink E., Gerdessen J.C., van Loon L.J., de Groot L.C. (2025). A Well-Balanced Vegan Diet Does not Compromise Daily Mixed Muscle Protein Synthesis Rates when Compared with an Omnivorous Diet in Active Older Adults: A Randomized Controlled Cross-Over Trial. J. Nutr..

[75] Hartman J.W., Tang J.E., Wilkinson S.B., Tarnopolsky M.A., Lawrence R.L., Fullerton A.V., Phillips S.M. (2007). Consumption of fat-free fluid milk after resistance exercise promotes greater lean mass accretion than does consumption of soy or carbohydrate in young, novice, male weightlifters. Am. J. Clin. Nutr..

[76] Chen S., Lin X., Ma J., Li M., Chen Y., Fang A.-P., Zhu H.-L. (2023). Dietary protein intake and changes in muscle mass measurements in community-dwelling middle-aged and older adults: A prospective cohort study. Clin. Nutr..

[77] Niklewicz A., Hannibal L., Warren M., Ahmadi K.R. (2024). A systematic review and meta-analysis of functional vitamin B12 status among adult vegans. Nutr. Bull..

[78] Kaviani M., Shaw K., Chilibeck P.D. (2020). Benefits of Creatine Supplementation for Vegetarians Compared to Omnivorous Athletes: A Systematic Review. Int. J. Environ. Res. Public Health.

[79] Blancquaert L., Baguet A., Bex T., Volkaert A., Everaert I., Delanghe J., Petrovic M., Vervaet C., De Henauw S., Constantin-Teodosiu D. (2018). Changing to a vegetarian diet reduces the body creatine pool in omnivorous women, but appears not to affect carnitine and carnosine homeostasis: A randomised trial. Br. J. Nutr..

[80] Lee Y.Q., Chia A., Sim X., van Dam R.M., Chong M.F.-F. (2025). Substituting animal protein foods with plant protein foods influences vitamin B12 and folate statuses in a multiethnic Asian population. Eur. J. Nutr..

